# Extending the Cooperative Phenotype: Assessing the Stability of Cooperation across Countries

**DOI:** 10.3389/fpsyg.2017.01990

**Published:** 2017-11-15

**Authors:** Amanda G. Reigstad, Eirik A. Strømland, Gustav Tinghög

**Affiliations:** ^1^Department of Economics, University of Bergen, Bergen, Norway; ^2^JEDILAB, Department of Management and Engineering, Linköping University, Linköping, Sweden; ^3^The National Center for Priority Setting in Health Care, Department of Medical and Health Sciences, Linköping University, Linköping, Sweden

**Keywords:** cooperation, social preferences, cooperative phenotype, prosocial behavior, trust

## Abstract

This paper studies whether individual cooperation is stable across settings and over time. Involving more than 7,000 subjects on two different continents, this study documents positive correlation in cooperative behavior across economic games in Norway, Sweden, Austria, and the United States. The game measures also correlate with a tendency to make deontological judgments in moral dilemmas, and display of general trust toward strangers. Using time-variation in the data, we test whether temporal stability of behavior is similar in the United States and Norway, and find similar stability estimates for both the American and Norwegian samples. The findings here provide further evidence of the existence of a stable behavioral inclination toward prosociality – a “cooperative phenotype,” as it has recently been termed. Also in line with previous research, we find that punishment and cooperation seem to be uncorrelated.

## Introduction

There is a substantial body of literature in the social sciences showing that people are willing to cooperate with others at personal cost.^1^ Theoretical models based on these experiments implicitly assume that the findings capture general insights about human behavior. However, few studies have explored to what extent the willingness to cooperate is stable across settings and over time. Such knowledge is important for our ability to extrapolate and systematically learn from experimental data.

This paper – based on a large sample of more than 7,000 individuals from Norway, Sweden, Austria, and the United States – shows that cooperative behavior is stable across settings and over time. The findings of this study support an implicit assumption underlying theories of social preferences – namely, that people have a domain-general and stable predisposition toward pro-sociality.

The idea of cooperation as a domain-general and stable personality trait has been dubbed the “cooperative phenotype” (Peysakhovich et al., 2014) to emphasize that the willingness to pay costs in order to benefit others is a stable and observable characteristic of an individual. While experimental games cannot pinpoint whether such stability of cooperation is genetic or environmental, they offer a promising opportunity to examine how widespread cooperative behavior is across contexts and over time. Moreover, experimental games can be used to quantify the strength of the relationship between different kinds of cooperative behavior in different settings.

Our study builds on previous work by Peysakhovich et al. (2014) that showed, using United States data from Amazon Mechanical Turk, that the same people tend to cooperate in different games.^2^ The findings here also support a growing set of studies on various aspects of the stability of pro-social behavior.^3^ Volk et al. (2012) showed that conditional contribution preferences are stable in a sample of lab students repeatedly re-invited to a lab over a period of 5 months. Another paper studying public-goods contributions in rural Vietnam found that cooperative behavior in that setting is temporally stable over long periods of time (Carlsson et al., 2014).^4^ Some other papers have also shown that different measures of pro-social behavior tend to be correlated across games (Yamagishi et al., 2013; Capraro et al., 2014; Böckler et al., 2016; Epstein et al., 2016).^5^

To our knowledge, this study is the first to explore inter-country differences in the stability of cooperation across settings and over time. This topic could be of substantial theoretical significance: If cooperation exhibits varying degrees of stability in different countries, it would have to be taken into account by models of social preferences (e.g., Rabin, 1993; Fehr and Schmidt, 1999; Bolton and Ockenfels, 2000; Falk and Fischbacher, 2006; Ellingsen and Johannesson, 2008). It is quite possible that cooperative behavior may exhibit varying degrees of stability in different countries; behavior in various economic games has been shown to display extensive cultural variation (Henrich et al., 2001; Herrmann et al., 2008).^6^ Falk et al. (2015) found correlations at the country level between survey measures of altruism, trust and reciprocity, but a study by Chuang and Schechter (2015) in Paraguay found much greater stability in survey measures than measures obtained from incentivized economic games. Differences in findings between survey measures and incentivized games point to the need for studying correlations in game behavior in addition to survey responses.

This paper proceeds as follows. First, we outline the data and methods. Correlations across different games are then analyzed, and then correlations between these measures and a survey measure of general trust are addressed. Finally, this paper examines temporal stability in the individual inclination to cooperate. Moreover, we compare time trends of cooperative behavior in the United States and Norway.

## Methods and Data Analysis

To minimize researcher degrees of freedom (Simmons et al., 2011), this paper adopted the analysis of Peysakhovich et al. (2014) as a benchmark. Thus, overall cross-sectional correlations in different game measures of cooperation are first considered. A stability measure of cooperative behavior over time is then constructed and compared between Norway and the United States. Finally, a formal test for systematic differences in cooperation trends between countries is conducted.^7^

### Norwegian Data: Methods and Procedures

Data from Norway was retrieved from a survey sent out to the “Norwegian Citizen Panel” (NCP), an online panel of a representative cross-section of the Norwegian population (Ivarsflaten et al., 2015). The survey sent out to the panel members included a series of incentivized economic games capturing different aspects of pro-social behavior: The Dictator Game (DG), the Public Goods Game (PGG) and the Prisoner’s Dilemma (PD). The survey also included a measure of general trust.

The DG is a game that measures altruism by asking a subject how much to share with a stranger. In the PGG, each individual in a group makes a choice about whether to contribute to a common pool. Finally, in the PD, each subject makes a binary choice about whether to cooperate with a randomly assigned partner. In both the PGG and the PD there are incentives to be selfish, but the group would benefit from cooperation. Following Peysakhovich et al. (2014), this study only included games in which no reputational incentives to cooperate were involved.

In addition to the economic games, the Norwegian Citizen Panel survey mainly includes general questions relating to politics and society. However, these survey questions are not included in our analysis, as they are not related to the main research question addressed in this study.

Panel members of the NCP are recruited through an invitation letter that is sent to a random sample of nearly 25,000 individuals listed in the Norwegian population registry with a current Norwegian address. The registry contains records of everyone born in Norway, as well as former and current inhabitants between the ages of 18 and 95. A total of 4,870 respondents registered for the NCP (Ivarsflaten et al., 2015). As some attrition is expected, the panel is refreshed at regular intervals through the sending out of new invitation letters. The final sample contains individuals from various age categories (such as young adults close to 20 years of age, or individuals above 60), a range of education categories (no education, only elementary school, higher education) and from all different geographical regions in Norway. It is worth noting that while the distribution of men and women in Norway is approximately 49.9 and 50.1%, in the NCP there are 50.3% men and 49.7% women.^8^

This paper employs data from December 2015 to April 2016, during which time a random subsample of the NCP participated in several economic games. The survey was conducted online. When subjects arrived at the part of the survey that involves economic games, they were informed that they would be making decisions that could result in real monetary payment. For each subsequent question they received separate instructions, and were required to make a choice by using a slider or filling out a number on the screen. After they made their choice and clicked to proceed with the survey, they were unable to go back to revise their response. Decisions were incentivized by informing participants that some decisions would be drawn for actual payment.

After the survey was concluded, researchers followed a double-blind process in which one researcher received the list of survey IDs and corresponding payment from the firm programming the survey, and then put payments into closed envelopes marked with the survey IDs. A second researcher received a list linking survey IDs to respondent names and addresses, and posted these in the mail without knowing anything about what each participant earned in the study.

**Table 1** summarizes the Norwegian data, the games included, and the time of the data collection.^9^ Brief summaries of the games included are provided below **Table 1**, while **Appendix B** provides detailed instructions for each game. The instructions were translated from Norwegian by the firm that provides the data (Skjervheim and Høgestøl, 2016). In each game, all participants were randomly assigned to play another participant without receiving any information about each other.

**Table 1:** Overview of the Norwegian data material.

Measure	Wave	*N*
Dictator Game	First	5,244
Public Goods Game	First	605
General Trust	First	5,429
Prisoner’s Dilemma	Second	1,079
Dictator Game w/Punishment	Second	1,060
Punishment	Second	243


The DG was conducted with 5,244 participants. In the DG, the “dictator” is assigned 2,000 Norwegian kroner^10^ (NOK), and asked to choose how much, in intervals of 100 NOK, to share with a randomly drawn individual. In the DG conducted in the NCP, two participants were randomly chosen to be either a dictator or a recipient. Participants were instructed that two participants from the total sample would randomly be assigned the role of either dictator or recipient. Hence, a random dictator rule was employed by which each person had an equal chance of dictating the result, and strategic considerations were eliminated.

The PGG was conducted with 605 participants. Each participant was allowed to choose how much of an endowment of 1,000 NOK^11^ he or she wished to contribute to a common pool. The amount contributed was then multiplied by a factor larger than one, and then evenly distributed among the group members. Consequently, in material terms it was payoff-maximizing to contribute zero regardless of what the other players decided. However, the group as a whole would benefit if all members contributed fully to the common pool. The particular PGG implemented in the NCP differed somewhat from the standard setup, in that one participant in each group of three was randomly excluded before the common pool was divided between the two remaining members of the group.^12^ Three subjects were randomly drawn to receive payment, and subjects were informed about the drawing prior to making any decisions.

The PD was conducted with 1,079 participants, who choose either to cooperate (“left”) or defect (“right”). If both players chose to cooperate, they received 800 NOK^13^ each. If one defected while the other cooperated, he or she received 1,200 NOK while the other got zero. Finally, if both defected, they each received 400 NOK^14^. Eight participants were randomly chosen to receive payment based on their decisions.

The Dictator Game with Punishment (DGP) was answered by 1,060 participants. The participant chose how much of 1,000 NOK to share with a randomly selected person, in preset amounts of 1, 250, 500, 750, or 1000. Afterward, participants could voluntarily decide how much to reduce the payoff to whoever kept the highest amount in a randomly selected sample of three participants. This “punishment” decision was voluntary, a fact that was stressed in the instructions to the participants. Despite being costless, only 367 chose to punish. In total, three participants were randomly drawn to receive payment based on their decisions in this game. The one who shared the least among these three was punished.^15^

Generalized Trust (GT) was measured in the first wave using the standard World Trust Survey question. Participants considered the claim, “Most people are to be trusted” on a scale from 0 to 10, where 0 is “can’t be careful enough” and 10 is “most people are to be trusted.” For the purposes of this study, this variable was converted to a scale from 0 to 5, in order to make the results directly comparable to Peysakhovich et al. (2014).^16^

### Results from Norwegian Data

**Table 2** displays pairwise (Pearson) correlations using the Norwegian data. All *p*-values are Bonferroni-corrected to adjust for multiple comparisons.

**Table 2:** Pearson correlations, Norway.

	DG	PGG	PD	DGP	Punishment
DG	1				
	(5244)				
PGG	0.2201^∗∗∗^	1			
	(605)	(605)			
PD	0.0451	0.1306^∗^	1		
	(915)	(456)	(1079)		
DGP	0.2541^∗∗∗^	0.2435^∗∗∗^	0.1191^∗∗∗^	1	
	(902)	(450)	(1026)	(1060)	
Punishment	0.0400	0.0466	0.0424	0.2698^∗∗∗^	1
	(211)	(95)	(241)	(241)	(243)


**N* in parentheses. ^∗^*p* < 0.10, ^∗∗^*p* < 0.05, ^∗∗∗^*p* < 0.01. *p*-values Bonferroni corrected to adjust for multiple comparisons. PGG, Public Goods Game; DG, Dictator Game; PD, Prisoner’s Dilemma; DGP, Dictator Game with Punishment; Punishment, Amount subtracted from player who kept most money.*

The different cooperation measures are significantly correlated. Notably, the correlation between DG giving and PGG contributions is quite large, with a correlation coefficient of 0.22. The correlation between the PD and other games is a bit smaller in magnitude, but qualitatively similar. The only two cooperation measures that are uncorrelated are the PD and the DG in the first wave, where a small positive and not statistically significant (*p* > 0.10) correlation is observed.^17^ Except for the DGP, significant correlations between the punishment measure and the measures of cooperation were not found.^18^ This result may have occurred because these choices were made sequentially by the same participants – first subjects chose an amount to share if drawn as dictator, and only afterward make a voluntary decision about whether and how much to punish the person who shares the least amount. The positive correlation between punishment and sharing in the DGP may reflect that those who choose to punish expect others to punish as well, and therefore perceive it to be in their self-interest to be pro-social in this specific game. This would also explain why the punishment measure does not correlate with any of the other measures of pro-sociality.

**FIGURE 1:**
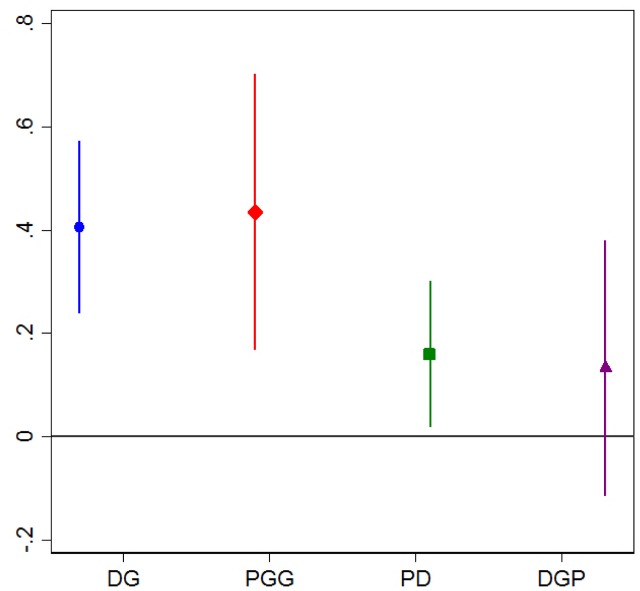
Regressions of generalized trust on behavioral measures (NCP data), with 95% confidence intervals. DG, Dictator Game; PGG, Public Goods Game; PD, Prisoner’s Dilemma; DGP, Dictator Game with Punishment.

Finally, we test whether the behavioral measures correlate with general trust. **Figure 1** visually displays the regression results from the regression of general trust on the different games included in the NCP. Except for the DGP, all behavioral measures correlate significantly with the measure of GT. This finding replicates results reported in Peysakhovich et al. (2014) that the PGG and DG are correlated with general trust.

### Swedish, Austrian and Additional American Data

The final data set used in this study to assess the domain-generality of cooperative behavior is a data set from Tinghög et al. (2013, 2016), featuring 1,101 individuals in Sweden, Austria, and the United States. The participants were given a PGG and a DG, as well as asked to render a decision on a Moral Dilemma (MD)^19^. In Sweden and Austria, data were collected in a lab setting, with student samples recruited through email. Data collection in the United States was conducted as a web survey, with subjects drawn from a sample of the adult American population included in the subject pool for Decision Research. **Table 3** displays separate and overall correlations.

**Table 3:** Pearson correlations (Sweden, Austria, and the United States).

	DG	PGG	MD
**SWE (*n* = 199)**			
DG	1		
PGG	0.1158	1	
MD	-0.2846***	0.0054	1
**United States (*n* = 582)**			
DG	1		
PGG	0.3089***	1	
MD	-0.0979	-0.0253	1
**AUS (*n* = 320)**			
DG	1		
PGG	0.1291	1	
MD	-0.0502	0.055	1
**Total (*n* = 1101)**			
DG	1		
PGG	0.2115***	1	
MD	-0.0835*	-0.0020	1


*^∗^*p* < 0.10, ^∗∗^*p* < 0.05, ^∗∗∗^*p* < 0.01. *p*–values Bonferroni corrected for 12 independent comparisons. DG, Dictator Game; PGG, Public Goods Game; MD, Moral Dilemma (The Trolley Problem).*

Overall giving in the DG is correlated with contributions to a public good (*p* < 0.01). With Bonferroni-corrections the results are not significant individually for Sweden and Austria, but this may result from this correction being overly conservative due to the assumption that the tests are independent. **Appendix A** displays uncorrected *p*-values. Here, the separate correlation coefficient for Austria is also statistically significant (*p* < 0.05). While the correlation between PGG giving and DG sharing is high and quantitatively similar for this American data set and the American data set in Peysakhovich et al. (2014), the estimated correlations between the PGG and DG are somewhat smaller in the Swedish and Austrian sample (although qualitatively similar).

### Stability of Cooperation Over Time in Norway and the United States

This paper now turns to the question of whether cooperative behavior is stable over time. The Norwegian data from the first wave was used to classify respondents into three cooperative types, following the classification procedure in Peysakhovich et al. (2014). Defectors are defined as those who share or contribute zero of their endowment in both the PGG and the DG. Cooperators are those who give or contribute more than 1% but less than 100% of their available total endowment. Finally, Super-cooperators contribute and share 100% in both games.

**FIGURE 2:**
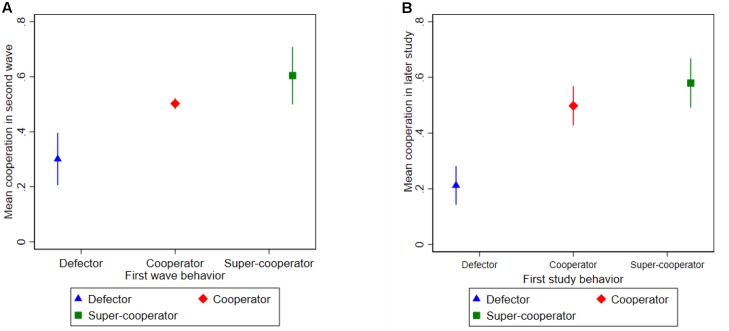
**(A)** Average cooperation in the NCP second wave for each type defined using first wave data (95% confidence intervals). **(B)** Coefficient plot from Peysakhovich et al. (2014).

An overall cooperation measure was created in the second wave by taking the mean of the DG and the PD decision. We then tested whether second-wave cooperation was associated with the pre-defined type measures. **Figure 2A** displays the results, and **Figure 2B** the corresponding findings reported in Peysakhovich et al. (2014). These results are robust to changing the cutoffs defining the cooperative types (see **Appendix A**).

Although the two waves in the NCP are measured approximately 4 months apart, a great degree of temporal stability was observed on the aggregate level.^20^ Defectors have much lower mean cooperation in the second wave than cooperators. In both Norway and the United States, cooperators exhibited roughly 50% mean cooperation, compared to approximately three-fifths for super-cooperators. Defectors in Norway cooperated about 30% in the second wave, while the corresponding figure in the United States was 21%. The results are strikingly similar between countries.

This study also defined a stability measure for each individual and compared it between countries. For each individual, a difference measure was defined by subtracting the average decision in the games played at time 1 from the average decision in the games played at time 2, and then taking the absolute value of this measure. This difference measure was then subtracted from one in order to obtain a stability measure. Thus, denoting by $\overset{¯}{y_{1i}}$ the average decision made at time 1 and by $\overset{¯}{y_{2i}}$ the average decision made at time 2, the stability measure is:

$$S_{i}=1-|\overset{¯}{y_{2i}}-\overset{¯}{y_{1i}}|$$

**FIGURE 3:**
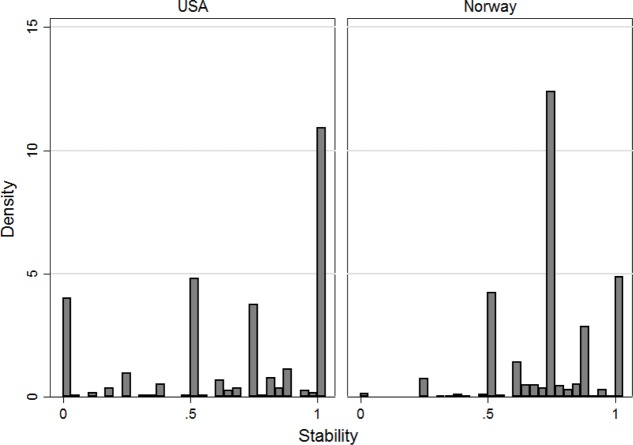
Histogram of stability measure in the United States and Norway.

This number is constructed to be in a unit interval [0, 1], as all variables have been normalized to one prior to defining it. The stability measure may therefore be interpreted as a measure of how similar decisions are between the two measurement waves. A measure of zero indicates that none of the decisions are similar, while a measure of one means that all decisions are identical. **Figure 3** displays the distribution of the stability measure in the United States and Norway using the data from the NCP and from Peysakhovich et al. (2014).

As **Figure 3** shows, whereas stability is high on average, there appears to be a substantial amount of individual variation. Overall, 70.8% of individual responses may be categorized as high stability (the stability measure is over 0.67), 7.5% of responses have low stability (measure below 0.33), and the rest have a medium level of stability. (See **Appendix Table A6** for details.) Thus, viewing the entire sample as a whole, there appears to be a high degree of stability both on the aggregate and on the individual level.

This paper now formally addresses inter-country differences in the temporal stability of cooperative behavior. Because this study did not have access to common demographic variables measured in both American and Norwegian data, it was not possible to directly control for demographic factors. However, by running a difference-in-differences regression model and controlling for individual fixed effects (FE), this analysis indirectly adjusted for all individual- and country-specific time-invariant effects that may influence cooperation. This was done by regressing the overall cooperation measure at time *t* for individual *i* on a constant, a dummy variable for wave 2, a country dummy and a set of individual-specific fixed effects that capture all time-invariant characteristics of individuals that influence cooperation. **Table 4** displays the regression results.

**Table 4:** Difference-in-differences results (Norway and the United States).

	(1)
Wave 2	-0.00354
	(0.0106)
United States^∗^Wave 2	-0.0501^∗^
	(0.0280)
Constant	0.483^∗∗∗^
	(0.00202)
Country FE	Yes
Subject FE	Yes
*N*	6960


*Cluster-robust standard errors in parentheses (clustered on subject). ^∗^*p* < 0.10, ^∗∗^*p* < 0.05, ^∗∗∗^*p* < 0.01. Reference group: Norway*

**Table 4** shows that mean cooperation, measured at 48.3%, is high in wave 1. There appears to be almost no change between the two waves; the coefficient on the wave 2 dummy is close to zero and not statistically significant. Moreover, while the difference-in-differences estimate indicates that cooperation decreases slightly more in the United States than in Norway over time (5 percentage points), this difference is not statistically significant at the 5% level. Thus, the null hypothesis that Norway and the United States display identical time-trends in cooperative behavior cannot be rejected.

It is worth noting that the regression results ultimately capture correlations. While all time-constant factors systematically influencing cooperation can be controlled for, other time-varying factors could systematically influence the time-trends in cooperation. For instance, the American sample features Amazon Mechanical Turk, while the Norwegian sample is from the NCP. There may be systematic differences over time in the demographic composition of these two samples.^21^

## Discussion and Conclusion

This study has shown that across several countries, different economic game measures of cooperation are positively correlated across settings. The data analyzed here also indicates that cooperative decisions are remarkably similar over time. Moreover, a comparison of Norway and the United States did not reject the null hypothesis of identical time-trends in cooperation over time. Overall, this analysis supports the hypothesis that human motivation is well captured by a “cooperative phenotype,” or a general behavioral disposition to pay costs to benefit others.

The result that prosocial behavior is on the overall level uncorrelated with punishment is in line with the findings reported in Peysakhovich et al. (2014). This empirical pattern seems inconsistent with theoretical models suggesting that altruism underlies the motivation to punish (e.g., Boyd et al., 2003; Hauert et al., 2007), and suggests that other potential mechanisms, such as anger, may explain punishing behavior (Jordan et al., 2016).

The findings of this study can contribute to further research into building general models of human motivation. The fact that the same people tend to cooperate in different games and over time suggests that one could build theoretical models of behavior in one setting and use the theory to predict cooperative behavior in a different context or time. As pro-social behavior exhibits a high degree of stability in Norway and the United States, there does not seem to be a pressing need to incorporate country-specific assumptions concerning the stability of other-regarding motivations.

While this sample was restricted to western, educated, industrialized, rich and democratic (“WEIRD”) societies (Henrich et al., 2010), the findings here are consistent with both a study conducted on correlation across games in Japan (Yamagishi et al., 2013) and another conducted in rural Vietnam (Volk et al., 2012). Viewed together with these studies, the findings here suggest that the “cooperative phenotype” persists across countries that are culturally quite different.

## Ethics Statement

We consulted the ethical review board for East Sweden to determine whether a formal approval of the committee was required. It was concluded that a formal assessment by the Ethics Committee was not necessary because the participants were given full-disclosure of the procedure (i.e., there was no deceit), participants received a payment proportionate to the task, the experimental procedure was non-invasive and the results were analyzed anonymously. Furthermore, the participants in all experiments were recruited online through our subject pools and voluntarily signed up for participation in the described experiments. They were informed participation was voluntary and anonymous. They were also informed that they could withdraw from the experiment at any time. All research at DIGSSCORE at the University of Bergen adheres to the ethical guidelines issued by the National Research Ethics Committee for Social Sciences and Humanities (NESH). Data from the Norwegian Citizen Panel are made available without restrictions for research purposes by the Norwegian Centre for Research data. In accordance with Norwegian data protection rules, only anonymous data are available to users. All experiments using economic games in the panel inform subjects that their participation is voluntary and anonymous. Furthermore, there is no use of deception.

## Author Contributions

All authors listed have made a substantial, direct and intellectual contribution to the work, and approved it for publication.

## Conflict of Interest Statement

The authors declare that the research was conducted in the absence of any commercial or financial relationships that could be construed as a potential conflict of interest.

## Figures and Tables

**FIGURE A1:**
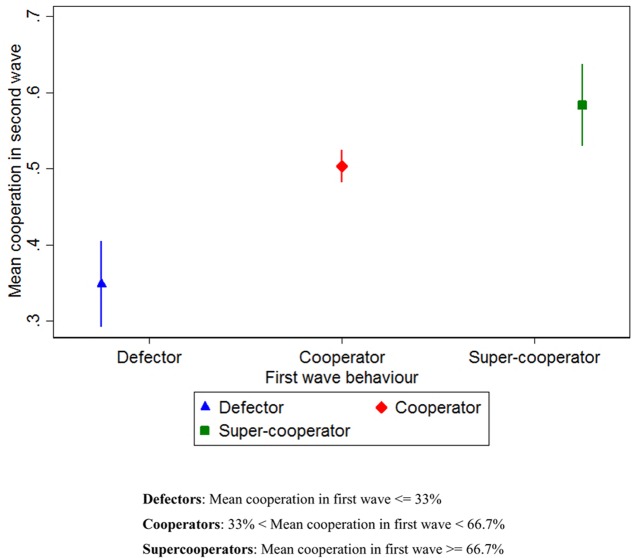
Coefficient plot, robustness check on findings in **Figure 2**.

## Footnotes

1 For summaries of this vast body of work, see e.g., Ledyard (1995), Chaudhuri (2011), and Engel (2011).

2 Given that this study used the same analysis criteria as Peysakhovich et al. (2014), the findings here can be considered to be out-of-sample tests on their results. Recent replication failures in the behavioral sciences (Open Science Collaboration, 2015; Camerer et al., 2016) underscore the importance of replicating previous work.

3 Other studies on the stability of other kinds of behavior include Cobb-Clark and Schurer (2012), which showed that the “Big Five” are stable over a time period of 4 years for working adults; Dasgupta et al. (2017), which tested for the stability of state-dependent preferences; and Meier and Sprenger (2015), which studied the stability of time preferences.

4 Blanco et al. (2011) studied whether inequity aversion predicts behavior over time across different games and on an individual level. They found overall stability, but not on an individual level.

5 Fischbacher et al. (2012) showed that different methods of measuring cooperation – the strategy method vs. direct responses – yield the same conclusions (the “strategy method” asks for choices for each hypothetical choice made by one’s partner in a game).

6 Although all four countries in this study are Western, industrialized nations, Norway and the United States are also quite different in a number of respects, such as income distribution (Almås et al., 2016).

7 **Appendix A**, **Tables A2**, **A3** reproduce the findings reported in Peysakhovich et al. (2014).

8 These figures may change somewhat over time as the panel recruits new respondents.

9 Generally, the attrition rate is low. In **Appendix A**, **Table A7** we report detailed data on attrition rates for each question included in the NCP data.

10 Approximately 240 USD.

11 Approximately 120 USD.

12 The rationale for this unconventional design is that this PGG was part of a larger experiment testing whether incentives to be part of a group shapes cooperation. This study used data on the control group that featured random exclusion of one of the three group members, which means that contributions do not determine group membership. Thus the optimal choice in material terms is unaffected by this design feature.

13 Approximately 100 USD.

14 Approximately 50 USD.

15 Because a coding error enabled invalid punishment responses (the program allowed for punishment levels outside of the possible range), only those punishment decisions that were valid in the analysis were included. Of 367 participants, the decisions of 243 remained. The results remained unchanged even if all punishment responses were included except for a single extreme outlier (with an invalid punishment level of 150,000).

16 The scale was converted by dividing the original scores in two, making a score of 10 equal to a score of 5, a score of 9 equal to a score of 4.5, and so forth. This has the benefit of preserving the same relationship between values as the original scale, while also making direct comparisons easier to interpret. By doing this, no information is lost. Moreover, the reported pairwise (Pearson) correlation coefficients are invariant to scale.

17 We do not conduct a factor-analysis, as this is not recommended when all correlations are weaker than 0.3 (Tabachnick et al., 2014, p. 667).

18 In **Appendix A**, we display alternative non-parametric Spearman correlations.

19 Whereas Tinghög et al. (2013, 2016) looks at the effects of time pressure and cognitive load on different economic decisions, this study pools these data and studies general correlation patterns across different games.

20 In the American data, the average time between the first and last decision was 124 days (Peysakhovich et al., 2014).

21 For instance, more women may drop out of the Norwegian survey between the two waves than in the United States sample. See Arechar et al. (2017) for a study on how participant characteristics vary over time on Amazon Mechanical Turk and Horton et al. (2011) for a general description of Amazon Mechanical Turk.

## APPENDIX A

**Table A1:** Spearman-correlations, Norway.

	DG	PGG	PD	DGP	Punishment
DG	1				
	(5244)				
PGG	0.2051^∗∗∗^	1			
	(605)	(605)			
PD	0.0415	0.1220^∗^	1		
	(915)	(456)	(1079)		
DGP	0.2396^∗∗∗^	0.2066^∗∗∗^	0.1250^∗∗∗^	1	
	(902)	(450)	(1026)	(1060)	
Punishment	-0.0042	0.0312	0.0758	0.3464^∗∗∗^	1
	(211)	(95)	(241)	(241)	(243)

**N* in parentheses. ^∗^*p* < 0.10, ^∗∗^*p* < 0.05, ^∗∗∗^*p* < 0.01. *p*-values Bonferroni corrected for multiple comparisons.*

**Table A2:** Summarizes the results in Peysakhovich et al. (2014), featuring approximately 1,400 individuals recruited on Amazon Mechanical Turk. The main takeaway is that different measures of cooperation are correlated, but unrelated to punishment. Pearson-correlations, United States.

	PGG	DG	TG1	TG2	UGMAO	PUND	TPP	AP
PGG	1							
DG	0.3887***	1						
TG1	0.3365***	0.3483***	1					
TG2	0.3861***	0.4948***	0.4927***	1				
UGMAO	0.1051	0.0631	0.0422	0.1179	1			
PUND	-0.0298	0.0658	0.0679	0.1472**	0.1809***	1		
TPP	0.0549	-0.0118	0.0631	0.0759	0.1593***	0.3550***	1	
AP	0.0202	0.0890	0.0281	0.0031	0.1048	0.1513**	0.0900	1

*^∗^*p* < 0.10, ^∗∗^*p* < 0.05, ^∗∗∗^*p* < 0.01. *p*-values Bonferroni corrected for multiple comparisons. PGG, Public Goods Game; DG, Dictator Game; TG1, First mover, Trust Game; TG2, Second mover, Trust Game; UGMAO, Minimum acceptable offer, Ultimatum Game; TPP, Third-party punishment; PUND, Second-party punishment, Prisoner’s Dilemma; AP, Two-player, sealed-bid All Pay Auction game.*

**Table A3:** Summarizes the results in Peysakhovich et al. (2014), featuring approximately 1,400 individuals recruited on Amazon Mechanical Turk. The main takeaway is that different measures of cooperation are correlated, but unrelated to punishment. Spearman-correlations, United States.

	PGG	DG	TG1	TG2	UGMAO	PUND	TPP	AP
PGG	1							
DG	0.4525***	1						
TG1	0.3477***	0.3449***	1					
TG2	0.4142***	0.4764***	0.4650***	1				
UGMAO	0.0951	0.0534	0.0515	0.1277	1			
PUND	0.0129	0.0669	0.0675	0.1171	0.1978***	1		
TPP	0.0947	0.0284	0.0793	0.1037	0.1627***	0.3991***	1	
AP	0.0455	0.0857	0.0246	-0.0282	0.1055	0.1658***	0.1180	1

*^∗^*p* < 0.10, ^∗∗^*p* < 0.05, ^∗∗∗^*p* < 0.01. *p*-values Bonferroni corrected for multiple comparisons. PGG, Public Goods Game; DG, Dictator Game; TG1, First mover, Trust Game; TG2, Second mover, Trust Game; UGMAO, Minimum acceptable offer, Ultimatum Game; TPP, Third-party punishment; PUND, Second-party punishment, Prisoner’s Dilemma; AP, Two-player, sealed-bid All Pay Auction game.*

**Table A4:** Uncorrected *p*-values.

	DG	Raw *p*	PGG	Raw *p*	MD
**SWE (*n* = 199)**					
DG	1				
PGG	0,1158	0,1034	1		
MD	-0.2846***	<0.0001	0,0054	0,9392	1
**United States (*n* = 582)**					
DG	1				
PGG	0.3089***	<0.0001	1		
MD	-0.0979**	0,0182	-0,0253	0,5427	1
**AUS (*n* = 320)**					
DG	1				
PGG	0.1291**	0.0209	1		
MD	-0,0502	0,3714	0,055	0,3271	1
**Total (*n* = 1101)**					
DG	1				
PGG	0.2115***	<0.0001	1		
MD	-0.0835^∗∗^	0,0056	-0,0020	0,9458	1

*^∗^*p* < 0.10, ^∗∗^*p* < 0.05, ^∗∗∗^*p* < 0.01.*

**Table A5:** Regression of general trust on behavioral measures (dependent variable ranges from 0 to 5).

	(1)	(2)	(3)	(4)
DG	0.405^∗∗∗^			
	(0.0854)			
PGG		0.435^∗∗∗^		
		(0.136)		
PD			0.160^∗∗^	
			(0.0722)	
DGP				0.133
				(0.126)
Constant	3.239^∗∗∗^	3.227^∗∗∗^	3.360^∗∗∗^	3.427^∗∗∗^
	(0.0438)	(0.0876)	(0.0577)	(0.0599)
*N*	5225	602	932	918

*Robust standard errors in parentheses. ^∗^*p* < 0.10, ^∗∗^*p* < 0.05, ^∗∗∗^*p* < 0.01*

**Table A6:** Degree of stability in the United States, Norway and overall.

Stability	United States	Norway	Overall
Low	18.6%	3.1%	7.5%
Middle	22.1%	21.6%	21.7%
High	59.3%	75.3%	70.8%
*N*	344	876	1220

*“Low” stability is defined as those with a stability measure less than 0.33, “High” is individuals with a stability measure higher than 0.67, and “Middle” is those in between.*

**Table A7:** Attrition rates by game.

Game	#	%
DG	207	3.8%
Trust	22	0.4%
PD	66	5.76%
PGG	40	6.2%
DGP	87	7.59%
Punishment	501	57.72%

*The high attrition rate for the “Punishment” variable may be because subjects were explicitly informed that they could voluntary opt out of this decision.*

## APPENDIX B: INSTRUCTIONS FOR THE GAMES INCLUDED IN THE ANALYSIS (TRANSLATED FROM NORWEGIAN)

### Dictator Game

As a participant in the Norwegian Citizen Panel you are being included in a draw for an extra monetary prize. Two people are drawn out: person A and person B. Person A receives 2,000 kroner and chooses how much is to be shared with person B. If I receive 2,000 kroner, I choose to give the following amount to the other person (between 0 and 2,000):

### Trust

Would you say that most people in general can be trusted, or do you think that one cannot be careful enough when dealing with others?

State your opinion on the scale below. This goes from 0 to 10, where 0 means “Cannot be careful enough” and 10 means “Most people can be trusted.”

### PGG – 1

Three participants in the Norwegian Citizen Panel are drawn to win an extra cash prize. These three are put together in a group. Every person in the group receives 1,000 kroner to begin with. The final amount of the cash prize depends on what choices the people in the group make in the decisions below. Each participant is to choose how much of his or her 1,000 kroner will be put into a joint kitty for the group. A person is drawn at random to be excluded from the group and loses his or her 1,000 kroner. The two remaining people’s contribution to the kitty is increased by 50 percent and then divided equally between the two. The final cash prize is each individual’s portion of the kitty and the money that was kept.

If you were drawn out to win 1,000 kroner, how much would you put into the joint kitty?

### PGG – 2

Three participants in the Norwegian Citizen Panel are drawn to win an extra cash prize. These three are put together in a group. Every person in the group receives 1,000 kroner to begin with. The final amount of the cash prize depends on what choices the people in the group make in the decisions below. Each participant is to choose how much of his or her 1,000 kroner will be put into a joint kitty for the group. A person is drawn at random to be excluded from the group, but keeps his or her 1,000 kroner. The two remaining people’s contribution to the kitty is increased by 50 percent and then divided equally between the two. The final cash prize is each individual’s portion of the kitty and the money that was kept.

If you were drawn out to win 1,000 kroner, how much would you put into the joint kitty?

### PD – 1

We will now ask you a question concerning taking a decision. We have drawn out four people who are paid based on the decision they take. You are completely anonymous and is to take only one decision.

You must choose to go either left or right. You will be randomly grouped with one other person who will take the same decision. How much you earn is dependent on the decisions you make: If you both choose to go left, you will receive 800 kroner each. If you choose to go left and the other person chooses to go right, you will receive 0 kroner and the other person will receive 1200 kroner. If you choose to go right and the other person chooses to go left, you will receive 1200 kroner and the other person will receive 0 kroner. If you both choose to go right, you will both receive 400 kroner each. Regardless of what the other person chooses, you stand to receive more by going right. The sum of the pay-out is biggest if both choose to go left.

### PD – 2

We will now ask you a question concerning taking a decision. We have drawn out four people who are paid based on the decision they take. You are completely anonymous and is to take only one decision.

You must choose to go either left or right. You will be randomly grouped with one other person who will take the same decision. How much you earn is dependent on the decisions you make: If you both choose to go left, you will receive 800 kroner each. If you choose to go left and the other person chooses to go right, you will receive 0 kroner and the other person will receive 1200 kroner. If you choose to go right and the other person chooses to go left, you will receive1200 kroner and the other person will receive 0 kroner. If you both choose to go right, you will both receive 400 kroner each. Regardless of what the other person chooses, you stand to receive more by going right. The sum of the pay-out is biggest if both choose to go left.

### DGP – 1

As a participant in the Norwegian Citizen Panel you and two other participants can be drawn out to win an extra cash prize of 1,000 kroner. If you are drawn out, you must all make two decisions. As decision 1, you must choose how much of the 1,000 kroner you would give a randomly selected member of the Norwegian Citizen Panel. As decision 2, you can choose to reduce the final prize of the one of the other two persons who has kept most of his or her 1,000 kroner.

Decision 1: If you were drawn out, how much of the 1,000 kroner would you give to a randomly selected member of the Norwegian Citizen Panel?

### DGP – 2

As a participant in the Norwegian Citizen Panel you and two other participants can be drawn out to win an extra cash prize of 1,000 kroner. If you are drawn out, you must all make two decisions. As decision 1, you must choose how much of the 1,000 kroner you would give a randomly selected member of the Norwegian Citizen Panel. As decision 2, you can choose to reduce the final prize of the one of the other two persons who has given least of his or her 1,000 kroner.

Decision 1: If you were drawn out, how much of the 1,000 kroner would you give to a randomly selected member of the Norwegian Citizen Panel?

### DGP – 3

As a participant in the Norwegian Citizen Panel you and two other participants can be drawn out to win an extra cash prize of 1,000 kroner. If you are drawn out, you must all make two decisions. As decision 1, you must choose how much of the 1,000 kroner you would give a randomly selected member of the Norwegian Citizen Panel. As decision 2, you can choose to reduce the final prize of the one of the other two persons who has kept most of his or her 1,000 kroner.

Decision 1: If you were drawn out, how much of the 1,000 kroner would you give to a randomly selected member of the Norwegian Citizen Panel?

### DGP – 4

As a participant in the Norwegian Citizen Panel you and two other participants can be drawn out to win an extra cash prize of 1,000 kroner. If you are drawn out, you must all make two decisions. As decision 1, you must choose how much of the 1,000 kroner you would give a randomly selected member of the Norwegian Citizen Panel. As decision 2, you can choose to reduce the final prize of the one of the other two persons who has given least of his or her 1,000 kroner.

Decision 1: If you were drawn out, how much of the 1,000 kroner would yougive to a randomly selected member of the Norwegian Citizen Panel.

### Punishment

For each question the respondent is presented with a sum of 0, 250, 500, 750, or 1000 kr

**1:**
Decision 2: In this decision, you can choose to reduce the final prize of the person who has kept most of his or her 1,000 kroner
Then I wish to reduce the prize to this person by the following amount:

**2:**
Decision 2: In this decision, you can choose to reduce the final prize of the person who has given least of his or her 1,000 kroner
Then I wish to reduce the prize to this person by the following amount:

**3:**
Decision 2: In this decision, you can choose to reduce the final prize of the person who has kept most of his or her 1,000 kroner.
Then I wish to reduce the prize to this person by the following amount:

**4:**
Decision 2: In this decision, you can choose to reduce the final prize of the person who has given least of his or her 1,000 kroner.
Then I wish to reduce the prize to this person by the following amount:
